# [4-(4-Meth­oxy­phen­yl)-8-oxo-3-(phenyl­selan­yl)spiro­[4.5]deca-3,6,9-trien-2-yl]methyl­cyanamide

**DOI:** 10.1107/S2414314620000784

**Published:** 2020-01-28

**Authors:** LaQuze L. Morris, Clarisa A. Alvarado, Julia M. Goncalves, Ravi P. Singh, Carl J. Lovely, Muhammed Yousufuddin

**Affiliations:** a Life and Health Sciences Department, University of North Texas at Dallas, 7400 University Hills Blvd, Dallas, TX 75241, USA; b Department of Chemistry and Biochemistry, University of Texas at Arlington, 701 S. Nedderman Dr., Arlington, TX 76019, USA; Howard University, USA

**Keywords:** crystal structure, cyanamide, propargyl­amines, spiro compounds, phenyl­selanyl derivatives

## Abstract

The title compound, C_25_H_22_N_2_O_2_Se, crystallizes in the space group *P*2_1_/*c* with one mol­ecule in the asymmetric unit. The compound was synthesized by the addition of phenyl­selenyl bromide to a cyanamide. The phenyl­selenyl portion and the cyano group, as well as the ketone functional group in the cyclo­hexa-2,5-dien-1-one portion of the structure, are disordered, with occupancy factors of 0.555 (14) and 0.445 (14).

## Abstract 2

The title compound, C_25_H_22_N_2_O_2_Se, crystallizes in the space group *P*2_1_/*c* with one mol­ecule in the asymmetric unit. The compound was synthesized by addition of phenyl­selenyl bromide to a cyanamide.

## Graphical abstract

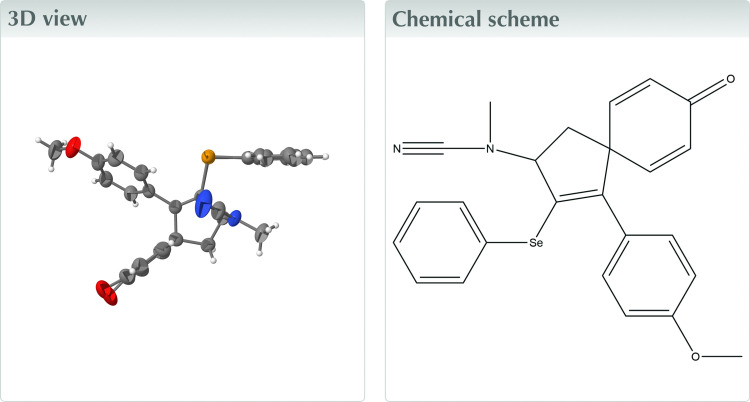

## Structure description

Previously, our group reported the synthesis of a spiro­cyclization compound (Singh *et al.*, 2016 ▸) while attempting to form a cyanamide (Yousufuddin *et al.*, 2018 ▸). We were able to show that the title compound could be synthesized by reacting the cyanamide with phenyl­selenyl bromide. We have since used spiro­cyclization of propargyl­amines (compounds that are related to cyanamides) to produce several thia­zolidines and thia­zolidones (Singh *et al.*, 2019 ▸).

**Figure 1:**
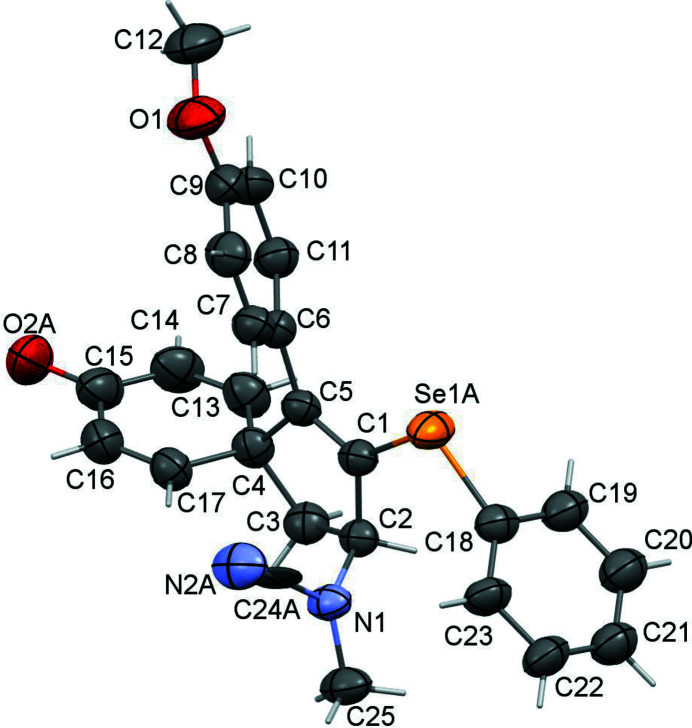
Molecular plot of title compound with ellipsoids drawn at 50% probability. Disordered portions are omitted for clarity.

The title compound crystallizes in the monoclinic space group *P*2_1_/*c*. There is one mol­ecule in the asymmetric unit yielding a *Z* value of 4 (Fig. 1 ▸).

The compound contains one phenyl­selanyl group that is disordered over two conformations, with occupancies of 0.555 (14) and 0.445 (14). The central five-membered ring is almost planar, with a maximum deviation for the C13 atom of only 0.133 (2) Å. In the spiro region of the mol­ecule, the dihedral angle between the central five-membered ring and the cyclo­hexa-2,5-dien-1-one unit is 88.22 (7)°. The dihedral angle between the central ring and the meth­oxy­phenyl group is only 55.98 (10)°, while the angle with the major component of the disordered phenyl­selanyl group is 88.6 (2)°. The Se1*A* atom is 1.883 (4) Å from C1 and 1.901 (7) Å from C18, while Se1*B* is 1.926 (5) Å from C1 and 1.920 (9) Å from C18. The C1—Se1*A*—C18 bond angle is 101.2 (4)° and the C1—Se1*B*—C18*A* bond angle is 98.2 (5)°.

## Synthesis and crystallization

The title compound was synthesized and crystallized following the procedure reported by our group (Singh *et al.*, 2016 ▸).

## Refinement

Crystal data, data collection and structure refinement details are summarized in Table 1 ▸. The refinement for the title compound indicated positional disorder at the phenyl ring attached to the Se atom and the cyano group as well as the ketone functional group in the cyclo­hexa-2,5-dien-1-one unit. These components were refined isotropically and the second component for each group was located in the resulting difference-Fourier map. The occupancies of these moieties were refined to a ratio of 0.555 (14):0.445 (14), with their anisotropic displacement parameters (ADP) treated with a combination of SIMU and DELU commands, which restrain the ADP values to be more reasonable.

## Supplementary Material

Crystal structure: contains datablock(s) I, global. DOI: 10.1107/S2414314620000784/bv4028sup1.cif

Structure factors: contains datablock(s) I. DOI: 10.1107/S2414314620000784/bv4028Isup2.hkl

Supporting information file. DOI: 10.1107/S2414314620000784/bv4028Isup3.cml

CCDC reference: 1979402

Additional supporting information:  crystallographic information; 3D view; checkCIF report

## Figures and Tables

**Table 1:** Experimental details

Crystal data	
Chemical formula	C_25_H_22_N_2_O_2_Se
*M*_r_	461.40
Crystal system, space group	Monoclinic, *P*2_1_/*c*
Temperature (K)	296
*a*, *b*, *c* (Å)	13.209 (7), 6.343 (3), 27.080 (14)
β (°)	103.356 (8)
*V* (Å^3^)	2207.4 (19)
*Z*	4
Radiation type	Mo *K*α
μ (mm^−1^)	1.72
Crystal size (mm)	0.70 × 0.18 × 0.10

Data collection	
Diffractometer	Bruker APEXII CCD
Absorption correction	Multi-scan (*SADABS*; Bruker, 2016 ▸)
*T*_min_, *T*_max_	0.466, 0.745
No. of measured, independent and observed [*I* > 2σ(*I*)] reflections	22486, 5498, 3448
*R*_int_	0.047
(sin θ/λ)_max_ (Å^−1^)	0.668

Refinement	
*R*[*F* ^2^ > 2σ(*F* ^2^)], *wR*(*F* ^2^), *S*	0.041, 0.108, 1.03
No. of reflections	5498
No. of parameters	322
No. of restraints	222
H-atom treatment	H-atom parameters constrained
Δρ_max_, Δρ_min_ (e Å^−3^)	0.34, −0.38

Computer programs: *APEX2* and *SAINT* (Bruker, 2016 ▸), *SHELXT2014* (Sheldrick, 2015*a* ▸), *SHELXL2018* (Sheldrick, 2015*b* ▸) and *SHELXTL* (Sheldrick, 2008 ▸).

## full crystallographic data

### Crystal data

**Table:**

C_25_H_22_N_2_O_2_Se	*F*(000) = 944
*M**_r_* = 461.40	*D*_x_ = 1.388 Mg m^−^^3^
Monoclinic, *P*2_1_/*c*	Mo *K*α radiation, λ = 0.71073 Å
*a* = 13.209 (7) Å	Cell parameters from 3960 reflections
*b* = 6.343 (3) Å	θ = 2.5–22.7°
*c* = 27.080 (14) Å	µ = 1.72 mm^−^^1^
β = 103.356 (8)°	*T* = 296 K
*V* = 2207.4 (19) Å^3^	Needle, colourless
*Z* = 4	0.70 × 0.18 × 0.10 mm

### Data collection

**Table:**

Bruker APEXII CCD diffractometer	3448 reflections with *I* > 2σ(*I*)
φ and ω scans	*R*_int_ = 0.047
Absorption correction: multi-scan (SADABS; Bruker, 2016)	θ_max_ = 28.3°, θ_min_ = 2.5°
*T*_min_ = 0.466, *T*_max_ = 0.745	*h* = −17→17
22486 measured reflections	*k* = −8→8
5498 independent reflections	*l* = −35→36

### Refinement

**Table:**

Refinement on *F*^2^	222 restraints
Least-squares matrix: full	Hydrogen site location: inferred from neighbouring sites
*R*[*F*^2^ > 2σ(*F*^2^)] = 0.041	H-atom parameters constrained
*wR*(*F*^2^) = 0.108	*w* = 1/[σ^2^(*F*_o_^2^) + (0.044*P*)^2^ + 0.3647*P*] where *P* = (*F*_o_^2^ + 2*F*_c_^2^)/3
*S* = 1.03	(Δ/σ)_max_ = 0.001
5498 reflections	Δρ_max_ = 0.34 e Å^−^^3^
322 parameters	Δρ_min_ = −0.38 e Å^−^^3^

### Special details

**Table:**

Geometry. All esds (except the esd in the dihedral angle between two l.s. planes) are estimated using the full covariance matrix. The cell esds are taken into account individually in the estimation of esds in distances, angles and torsion angles; correlations between esds in cell parameters are only used when they are defined by crystal symmetry. An approximate (isotropic) treatment of cell esds is used for estimating esds involving l.s. planes.
Refinement. The H atoms were included in calculated positions and refined using a riding model, with C—H = 0.93, 0.96, 0.97, and 0.98 Å for aromatic, methyl, methylene, and terminal H atoms, respectively, and with *U*_iso_(H) = 1.2*U*_eq_(C) and 1.5*U*_eq_(*C*-methyl).

### Fractional atomic coordinates and isotropic or equivalent isotropic displacement parameters (Å^2^)

**Table:**

	*x*	*y*	*z*	*U*_iso_*/*U*_eq_	Occ. (<1)
Se1A	0.6308 (3)	0.4941 (10)	0.26771 (16)	0.0642 (7)	0.555 (14)
Se1B	0.6377 (4)	0.5228 (12)	0.2657 (2)	0.0694 (9)	0.445 (14)
O1	0.43190 (15)	1.1265 (3)	0.40821 (9)	0.0857 (6)	
O2A	0.868 (2)	0.680 (5)	0.5648 (13)	0.090 (4)	0.555 (14)
O2B	0.884 (3)	0.623 (6)	0.5682 (15)	0.098 (6)	0.445 (14)
N1	0.90074 (14)	0.4281 (3)	0.31172 (8)	0.0486 (5)	
N2	0.9080 (2)	0.8146 (4)	0.31572 (12)	0.0968 (10)	
C1	0.72698 (16)	0.4392 (3)	0.32939 (9)	0.0433 (5)	
C2	0.82397 (16)	0.3120 (3)	0.33276 (8)	0.0429 (5)	
H2	0.806314	0.179735	0.314056	0.051*	
C3	0.85901 (19)	0.2646 (4)	0.38949 (9)	0.0529 (6)	
H3A	0.934144	0.274277	0.400499	0.063*	
H3B	0.837873	0.123490	0.396611	0.063*	
C4	0.80622 (18)	0.4317 (4)	0.41768 (9)	0.0482 (6)	
C5	0.71565 (17)	0.5081 (3)	0.37431 (9)	0.0440 (5)	
C6	0.63660 (17)	0.6620 (4)	0.38302 (8)	0.0459 (5)	
C7	0.62917 (18)	0.8586 (4)	0.35983 (9)	0.0543 (6)	
H7	0.672234	0.890191	0.338088	0.065*	
C8	0.5593 (2)	1.0078 (4)	0.36838 (12)	0.0620 (7)	
H8	0.554882	1.138108	0.352278	0.074*	
C9	0.4961 (2)	0.9627 (4)	0.40089 (11)	0.0582 (7)	
C10	0.49946 (19)	0.7697 (4)	0.42348 (9)	0.0602 (7)	
H10	0.455269	0.739109	0.444734	0.072*	
C11	0.56992 (19)	0.6186 (4)	0.41439 (9)	0.0555 (6)	
H11	0.572089	0.486786	0.429638	0.067*	
C12	0.3628 (3)	1.0895 (6)	0.44033 (13)	0.0939 (10)	
H12D	0.313342	0.983074	0.425567	0.141*	
H12E	0.326669	1.217622	0.444198	0.141*	
H12F	0.401554	1.042867	0.472958	0.141*	
C13	0.7701 (2)	0.3312 (4)	0.46039 (10)	0.0599 (7)	
H13	0.728124	0.212131	0.453276	0.072*	
C14	0.7940 (2)	0.4009 (5)	0.50789 (10)	0.0708 (8)	
H14	0.769986	0.326985	0.532572	0.085*	
C15	0.8565 (2)	0.5889 (6)	0.52255 (11)	0.0713 (8)	
C16	0.8959 (2)	0.6902 (4)	0.48293 (10)	0.0628 (7)	
H16	0.937462	0.809446	0.490864	0.075*	
C17	0.87484 (18)	0.6182 (4)	0.43561 (9)	0.0532 (6)	
H17	0.904369	0.687637	0.412135	0.064*	
C18	0.6898 (7)	0.3385 (11)	0.2213 (4)	0.0502 (15)	0.555 (14)
C19	0.6434 (6)	0.1406 (12)	0.2145 (3)	0.0605 (15)	0.555 (14)
H19A	0.592557	0.104961	0.231710	0.073*	0.555 (14)
C20	0.6731 (7)	−0.0041 (8)	0.1819 (3)	0.0690 (16)	0.555 (14)
H20A	0.642134	−0.136467	0.177392	0.083*	0.555 (14)
C21	0.7492 (6)	0.0492 (13)	0.1562 (2)	0.0632 (17)	0.555 (14)
H21A	0.769056	−0.047586	0.134438	0.076*	0.555 (14)
C22	0.7955 (5)	0.2471 (15)	0.1630 (3)	0.0640 (16)	0.555 (14)
H22A	0.846402	0.282725	0.145803	0.077*	0.555 (14)
C23	0.7658 (7)	0.3917 (11)	0.1956 (3)	0.0584 (14)	0.555 (14)
H23A	0.796827	0.524156	0.200121	0.070*	0.555 (14)
C18A	0.6849 (10)	0.3222 (15)	0.2228 (5)	0.0531 (18)	0.445 (14)
C19A	0.6599 (8)	0.1141 (15)	0.2091 (4)	0.0598 (17)	0.445 (14)
H19B	0.612225	0.041799	0.223214	0.072*	0.445 (14)
C20A	0.7062 (8)	0.0142 (10)	0.1743 (4)	0.0644 (19)	0.445 (14)
H20B	0.689498	−0.125007	0.165074	0.077*	0.445 (14)
C21A	0.7775 (7)	0.1223 (17)	0.1532 (3)	0.0627 (19)	0.445 (14)
H21B	0.808463	0.055477	0.129846	0.075*	0.445 (14)
C22A	0.8025 (6)	0.3304 (18)	0.1669 (4)	0.0675 (18)	0.445 (14)
H22B	0.850157	0.402767	0.152757	0.081*	0.445 (14)
C23A	0.7562 (9)	0.4304 (12)	0.2017 (5)	0.0629 (18)	0.445 (14)
H23B	0.772885	0.569577	0.210896	0.076*	0.445 (14)
C24	0.9049 (2)	0.6355 (4)	0.31394 (10)	0.0572 (6)	
C25	0.9865 (2)	0.3112 (4)	0.29936 (11)	0.0645 (7)	
H25A	1.026422	0.403648	0.283148	0.097*	
H25B	0.959357	0.197260	0.276855	0.097*	
H25C	1.030196	0.255783	0.329927	0.097*	

### Atomic displacement parameters (Å^2^)

**Table:**

	*U*^11^	*U*^22^	*U*^33^	*U*^12^	*U*^13^	*U*^23^
Se1A	0.0461 (8)	0.0904 (17)	0.0530 (12)	0.0220 (7)	0.0050 (6)	−0.0105 (10)
Se1B	0.081 (2)	0.0812 (14)	0.0464 (10)	0.0381 (14)	0.0162 (11)	0.0066 (9)
O1	0.0773 (14)	0.0701 (13)	0.1199 (17)	0.0161 (11)	0.0439 (13)	−0.0128 (12)
O2A	0.075 (7)	0.140 (10)	0.057 (4)	0.008 (5)	0.018 (4)	−0.023 (6)
O2B	0.072 (10)	0.170 (19)	0.055 (7)	−0.012 (11)	0.021 (7)	−0.039 (11)
N1	0.0476 (11)	0.0361 (10)	0.0676 (13)	0.0036 (8)	0.0250 (10)	−0.0006 (9)
N2	0.131 (2)	0.0393 (14)	0.149 (3)	−0.0025 (13)	0.092 (2)	0.0027 (15)
C1	0.0399 (12)	0.0427 (12)	0.0487 (13)	0.0004 (9)	0.0134 (10)	0.0010 (10)
C2	0.0422 (12)	0.0379 (12)	0.0502 (13)	0.0003 (9)	0.0142 (10)	0.0010 (10)
C3	0.0530 (14)	0.0495 (14)	0.0560 (14)	0.0052 (11)	0.0124 (11)	0.0039 (11)
C4	0.0495 (14)	0.0508 (13)	0.0455 (13)	−0.0051 (11)	0.0136 (10)	0.0041 (11)
C5	0.0399 (11)	0.0464 (13)	0.0482 (12)	−0.0028 (10)	0.0149 (10)	−0.0007 (11)
C6	0.0435 (13)	0.0499 (13)	0.0455 (12)	−0.0042 (10)	0.0125 (10)	−0.0078 (11)
C7	0.0504 (14)	0.0523 (15)	0.0638 (15)	−0.0046 (11)	0.0205 (12)	−0.0046 (12)
C8	0.0603 (16)	0.0481 (15)	0.0793 (19)	−0.0019 (12)	0.0196 (14)	−0.0024 (13)
C9	0.0516 (15)	0.0561 (16)	0.0687 (17)	−0.0022 (12)	0.0180 (13)	−0.0166 (13)
C10	0.0517 (15)	0.0740 (18)	0.0607 (16)	−0.0035 (13)	0.0248 (12)	−0.0078 (14)
C11	0.0563 (15)	0.0571 (15)	0.0568 (15)	−0.0006 (12)	0.0208 (12)	−0.0024 (12)
C12	0.072 (2)	0.116 (3)	0.101 (3)	0.024 (2)	0.0358 (19)	−0.025 (2)
C13	0.0585 (16)	0.0653 (16)	0.0566 (16)	−0.0089 (13)	0.0145 (12)	0.0097 (13)
C14	0.0679 (18)	0.099 (2)	0.0490 (16)	−0.0049 (17)	0.0210 (13)	0.0127 (16)
C15	0.0529 (16)	0.108 (2)	0.0532 (17)	0.0005 (16)	0.0122 (13)	−0.0099 (17)
C16	0.0529 (15)	0.0700 (17)	0.0645 (17)	−0.0075 (13)	0.0120 (12)	−0.0131 (14)
C17	0.0487 (14)	0.0558 (15)	0.0551 (15)	−0.0056 (11)	0.0119 (11)	0.0018 (12)
C18	0.045 (3)	0.064 (3)	0.041 (3)	0.008 (3)	0.009 (2)	0.003 (3)
C19	0.059 (3)	0.070 (3)	0.054 (3)	0.011 (3)	0.017 (2)	−0.004 (3)
C20	0.068 (4)	0.080 (3)	0.061 (3)	0.009 (3)	0.018 (3)	−0.009 (3)
C21	0.066 (4)	0.074 (4)	0.053 (3)	0.008 (3)	0.021 (3)	−0.006 (3)
C22	0.066 (3)	0.075 (4)	0.054 (3)	0.014 (3)	0.020 (2)	−0.011 (3)
C23	0.057 (3)	0.075 (3)	0.049 (3)	0.007 (3)	0.025 (2)	−0.003 (3)
C18A	0.051 (3)	0.063 (3)	0.044 (3)	0.010 (3)	0.009 (3)	−0.001 (3)
C19A	0.063 (3)	0.064 (3)	0.055 (3)	0.008 (3)	0.018 (3)	−0.007 (3)
C20A	0.070 (4)	0.071 (3)	0.055 (3)	0.010 (3)	0.021 (3)	−0.007 (3)
C21A	0.066 (4)	0.070 (4)	0.054 (3)	0.009 (3)	0.018 (3)	−0.008 (3)
C22A	0.073 (3)	0.076 (4)	0.057 (3)	0.008 (3)	0.020 (3)	−0.009 (4)
C23A	0.062 (3)	0.076 (3)	0.054 (3)	0.014 (3)	0.019 (3)	−0.008 (3)
C24	0.0641 (16)	0.0477 (16)	0.0697 (17)	0.0007 (12)	0.0362 (13)	0.0036 (12)
C25	0.0585 (16)	0.0543 (15)	0.091 (2)	0.0049 (12)	0.0378 (14)	−0.0049 (14)

### Geometric parameters (Å, º)

**Table:**

Se1A—C1	1.883 (4)	C12—H12F	0.9600
Se1A—C18	1.901 (6)	C13—C14	1.328 (4)
Se1B—C18A	1.921 (9)	C13—H13	0.9300
Se1B—C1	1.926 (5)	C14—C15	1.452 (4)
O1—C9	1.384 (3)	C14—H14	0.9300
O1—C12	1.418 (4)	C15—C16	1.447 (4)
O2A—C15	1.26 (3)	C16—C17	1.328 (3)
O2B—C15	1.22 (4)	C16—H16	0.9300
N1—C24	1.318 (3)	C17—H17	0.9300
N1—C25	1.456 (3)	C18—C19	1.3900
N1—C2	1.470 (3)	C18—C23	1.3900
N2—C24	1.137 (3)	C19—C20	1.3900
C1—C5	1.333 (3)	C19—H19A	0.9300
C1—C2	1.499 (3)	C20—C21	1.3900
C2—C3	1.528 (3)	C20—H20A	0.9300
C2—H2	0.9800	C21—C22	1.3900
C3—C4	1.562 (3)	C21—H21A	0.9300
C3—H3A	0.9700	C22—C23	1.3900
C3—H3B	0.9700	C22—H22A	0.9300
C4—C13	1.492 (3)	C23—H23A	0.9300
C4—C17	1.501 (3)	C18A—C19A	1.3900
C4—C5	1.548 (3)	C18A—C23A	1.3900
C5—C6	1.488 (3)	C19A—C20A	1.3900
C6—C11	1.385 (3)	C19A—H19B	0.9300
C6—C7	1.389 (3)	C20A—C21A	1.3900
C7—C8	1.378 (3)	C20A—H20B	0.9300
C7—H7	0.9300	C21A—C22A	1.3900
C8—C9	1.376 (4)	C21A—H21B	0.9300
C8—H8	0.9300	C22A—C23A	1.3900
C9—C10	1.364 (4)	C22A—H22B	0.9300
C10—C11	1.397 (3)	C23A—H23B	0.9300
C10—H10	0.9300	C25—H25A	0.9600
C11—H11	0.9300	C25—H25B	0.9600
C12—H12D	0.9600	C25—H25C	0.9600
C12—H12E	0.9600		
			
C1—Se1A—C18	101.2 (4)	C4—C13—H13	117.9
C18A—Se1B—C1	98.1 (5)	C13—C14—C15	122.1 (3)
C9—O1—C12	117.8 (2)	C13—C14—H14	119.0
C24—N1—C25	119.50 (19)	C15—C14—H14	119.0
C24—N1—C2	120.49 (18)	O2B—C15—C16	127 (2)
C25—N1—C2	118.50 (18)	O2A—C15—C16	118.7 (17)
C5—C1—C2	113.3 (2)	O2B—C15—C14	116 (2)
C5—C1—Se1A	123.6 (2)	O2A—C15—C14	124.6 (17)
C2—C1—Se1A	123.1 (2)	C16—C15—C14	116.2 (2)
C5—C1—Se1B	123.5 (3)	C17—C16—C15	122.1 (3)
C2—C1—Se1B	122.7 (2)	C17—C16—H16	118.9
N1—C2—C1	111.31 (17)	C15—C16—H16	118.9
N1—C2—C3	114.34 (18)	C16—C17—C4	124.0 (2)
C1—C2—C3	102.90 (17)	C16—C17—H17	118.0
N1—C2—H2	109.4	C4—C17—H17	118.0
C1—C2—H2	109.4	C19—C18—C23	120.0
C3—C2—H2	109.4	C19—C18—Se1A	108.8 (5)
C2—C3—C4	106.98 (18)	C23—C18—Se1A	131.2 (5)
C2—C3—H3A	110.3	C20—C19—C18	120.0
C4—C3—H3A	110.3	C20—C19—H19A	120.0
C2—C3—H3B	110.3	C18—C19—H19A	120.0
C4—C3—H3B	110.3	C19—C20—C21	120.0
H3A—C3—H3B	108.6	C19—C20—H20A	120.0
C13—C4—C17	111.3 (2)	C21—C20—H20A	120.0
C13—C4—C5	113.1 (2)	C20—C21—C22	120.0
C17—C4—C5	107.56 (19)	C20—C21—H21A	120.0
C13—C4—C3	110.4 (2)	C22—C21—H21A	120.0
C17—C4—C3	112.87 (19)	C23—C22—C21	120.0
C5—C4—C3	101.28 (18)	C23—C22—H22A	120.0
C1—C5—C6	126.2 (2)	C21—C22—H22A	120.0
C1—C5—C4	111.1 (2)	C22—C23—C18	120.0
C6—C5—C4	122.2 (2)	C22—C23—H23A	120.0
C11—C6—C7	117.8 (2)	C18—C23—H23A	120.0
C11—C6—C5	122.4 (2)	C19A—C18A—C23A	120.0
C7—C6—C5	119.7 (2)	C19A—C18A—Se1B	134.5 (7)
C8—C7—C6	121.3 (2)	C23A—C18A—Se1B	105.5 (7)
C8—C7—H7	119.3	C20A—C19A—C18A	120.0
C6—C7—H7	119.3	C20A—C19A—H19B	120.0
C9—C8—C7	119.6 (2)	C18A—C19A—H19B	120.0
C9—C8—H8	120.2	C21A—C20A—C19A	120.0
C7—C8—H8	120.2	C21A—C20A—H20B	120.0
C10—C9—C8	120.8 (2)	C19A—C20A—H20B	120.0
C10—C9—O1	124.5 (2)	C20A—C21A—C22A	120.0
C8—C9—O1	114.7 (2)	C20A—C21A—H21B	120.0
C9—C10—C11	119.4 (2)	C22A—C21A—H21B	120.0
C9—C10—H10	120.3	C21A—C22A—C23A	120.0
C11—C10—H10	120.3	C21A—C22A—H22B	120.0
C6—C11—C10	121.0 (2)	C23A—C22A—H22B	120.0
C6—C11—H11	119.5	C22A—C23A—C18A	120.0
C10—C11—H11	119.5	C22A—C23A—H23B	120.0
O1—C12—H12D	109.5	C18A—C23A—H23B	120.0
O1—C12—H12E	109.5	N2—C24—N1	179.7 (4)
H12D—C12—H12E	109.5	N1—C25—H25A	109.5
O1—C12—H12F	109.5	N1—C25—H25B	109.5
H12D—C12—H12F	109.5	H25A—C25—H25B	109.5
H12E—C12—H12F	109.5	N1—C25—H25C	109.5
C14—C13—C4	124.2 (3)	H25A—C25—H25C	109.5
C14—C13—H13	117.9	H25B—C25—H25C	109.5
			
C18—Se1A—C1—C5	−176.5 (3)	C12—O1—C9—C10	−1.8 (4)
C18—Se1A—C1—C2	3.1 (5)	C12—O1—C9—C8	178.5 (3)
C24—N1—C2—C1	30.3 (3)	C8—C9—C10—C11	1.6 (4)
C25—N1—C2—C1	−163.8 (2)	O1—C9—C10—C11	−178.0 (2)
C24—N1—C2—C3	−85.8 (3)	C7—C6—C11—C10	−1.7 (4)
C25—N1—C2—C3	80.2 (3)	C5—C6—C11—C10	177.3 (2)
C5—C1—C2—N1	−109.0 (2)	C9—C10—C11—C6	0.3 (4)
Se1A—C1—C2—N1	71.3 (3)	C17—C4—C13—C14	1.3 (4)
Se1B—C1—C2—N1	63.7 (4)	C5—C4—C13—C14	−119.9 (3)
C5—C1—C2—C3	13.9 (2)	C3—C4—C13—C14	127.4 (3)
Se1A—C1—C2—C3	−165.8 (3)	C4—C13—C14—C15	1.8 (5)
Se1B—C1—C2—C3	−173.4 (3)	C13—C14—C15—O2B	−171.4 (16)
N1—C2—C3—C4	100.3 (2)	C13—C14—C15—O2A	168.6 (13)
C1—C2—C3—C4	−20.6 (2)	C13—C14—C15—C16	−3.2 (4)
C2—C3—C4—C13	139.8 (2)	O2B—C15—C16—C17	168.1 (17)
C2—C3—C4—C17	−95.0 (2)	O2A—C15—C16—C17	−171.0 (13)
C2—C3—C4—C5	19.7 (2)	C14—C15—C16—C17	1.3 (4)
C2—C1—C5—C6	170.7 (2)	C15—C16—C17—C4	2.0 (4)
Se1A—C1—C5—C6	−9.6 (4)	C13—C4—C17—C16	−3.2 (3)
Se1B—C1—C5—C6	−1.9 (4)	C5—C4—C17—C16	121.2 (3)
C2—C1—C5—C4	−1.2 (3)	C3—C4—C17—C16	−128.0 (3)
Se1A—C1—C5—C4	178.5 (3)	C23—C18—C19—C20	0.0
Se1B—C1—C5—C4	−173.9 (3)	Se1A—C18—C19—C20	179.8 (7)
C13—C4—C5—C1	−129.8 (2)	C18—C19—C20—C21	0.0
C17—C4—C5—C1	106.9 (2)	C19—C20—C21—C22	0.0
C3—C4—C5—C1	−11.7 (2)	C20—C21—C22—C23	0.0
C13—C4—C5—C6	57.9 (3)	C21—C22—C23—C18	0.0
C17—C4—C5—C6	−65.4 (3)	C19—C18—C23—C22	0.0
C3—C4—C5—C6	175.99 (19)	Se1A—C18—C23—C22	−179.8 (8)
C1—C5—C6—C11	125.9 (3)	C23A—C18A—C19A—C20A	0.0
C4—C5—C6—C11	−63.1 (3)	Se1B—C18A—C19A—C20A	176.7 (11)
C1—C5—C6—C7	−55.1 (3)	C18A—C19A—C20A—C21A	0.0
C4—C5—C6—C7	115.9 (2)	C19A—C20A—C21A—C22A	0.0
C11—C6—C7—C8	1.2 (4)	C20A—C21A—C22A—C23A	0.0
C5—C6—C7—C8	−177.8 (2)	C21A—C22A—C23A—C18A	0.0
C6—C7—C8—C9	0.6 (4)	C19A—C18A—C23A—C22A	0.0
C7—C8—C9—C10	−2.1 (4)	Se1B—C18A—C23A—C22A	−177.6 (8)
C7—C8—C9—O1	177.6 (2)		

### Hydrogen-bond geometry (Å, º)

**Table:**

*D*—H···*A*	*D*—H	H···*A*	*D*···*A*	*D*—H···*A*
C2—H2···N2^i^	0.98	2.67	3.411 (4)	132
C3—H3*A*···O2*A*^ii^	0.97	2.58	3.55 (3)	176
C3—H3*A*···O2*B*^ii^	0.97	2.44	3.40 (4)	168
C12—H12*E*···O2*A*^iii^	0.96	2.61	3.36 (3)	135
C19—H19*A*···Se1*A*^iv^	0.93	3.04	3.876 (8)	151

Symmetry codes: (i) *x*, *y*−1, *z*; (ii) −*x*+2, −*y*+1, −*z*+1; (iii) −*x*+1, −*y*+2, −*z*+1; (iv) −*x*+1, *y*−1/2, −*z*+1/2.
